# Improving Care & Outcomes for People with the Chronic Disease of Obesity using the KAER Framework

**DOI:** 10.1093/geroni/igaf122.4380

**Published:** 2025-12-31

**Authors:** Elana Kieffer Blass, Jennifer Pettis, Lisa McGuire, Ophira Bansal

**Affiliations:** Gerontological Society of America, Washington, District of Columbia, United States; Gerontological Society of America, Washington, District of Columbia, United States; Gerontological Society of America, Atlanta, Georgia, United States; Gerontology Society of America, Washington, District of Columbia, United States

## Abstract

Obesity prevalence for adults aged 65 years old and older has nearly doubled from 22% in 1988 to 42% in 2020. This increase has brought much needed attention to the chronic disease of obesity in older adults and their unique needs when it comes to discussions about and treatment options for overweight and obesity. The Gerontological Society of America (GSA) KAER Toolkit for the Management of Obesity in Older Adults provides a framework for primary care providers to help older people with obesity address this chronic health condition. The newly updated web-based toolkit utilizes the KAER framework to help providers of older adults with recognize and care for obesity in their patients. The four steps of the KAER framework—Kickstart, Assess, Evaluate, and Refer (KAER)—were initially formulated to improve health-related outcomes for people with dementia. Building on the success of this initial work, the KAER framework has been leveraged to help primary care providers improve outcomes for other conditions, including obesity. This presentation will demonstrate how providers can help older adults with obesity improve their health, function, and quality of life with practical approaches, educational resources, practice pearls, and validated clinical tools, that teams can immediately integrate into their workflow.

